# Health and fitness trends in Southern Europe for 2023: A cross-sectional survey

**DOI:** 10.3934/publichealth.2023028

**Published:** 2023-05-09

**Authors:** Alexios Batrakoulis, Oscar L Veiga, Susana Franco, Ewan Thomas, Antonios Alexopoulos, Manel Valcarce-Torrente, Rita Santos-Rocha, Fatima Ramalho, Andrea Di Credico, Daniela Vitucci, Liliana Ramos, Vera Simões, Alejandro Romero-Caballero, Isabel Vieira, Annamaria Mancini, Antonino Bianco

**Affiliations:** 1 Department of Physical Education and Sport Science, University of Thessaly, Trikala, Greece; 2 Department of Physical Education, Sports and Human Movement, Autonomous University of Madrid, Madrid, Spain; 3 Sport Sciences School of Rio Maior, Polytechnic Institute of Santarém, Rio Maior, Portugal; 4 Quality of Life Research Centre (CIEQV), Rio Maior, Portugal; 5 Department of Psychology, Educational Science and Human Movement, University of Palermo, Palermo, Italy; 6 Department of Life Sciences, European University Cyprus, Nicosia, Cyprus; 7 Department of Business Management, Valencian International University, Valencia, Spain; 8 Interdisciplinary Centre for the Study of Human Performance (CIPER), Lisbon, Portugal; 9 Department of Medicine and Aging Sciences, D'Annunzio University of Chieti-Pescara, Chieti, Italy; 10 Department of Movement Sciences and Wellness, University Parthenope, Napoli, Italy; 11 Department of Physical Education, Camilo José Cela University, Madrid, Spain

**Keywords:** Southern Europe, fitness survey, trends, top programs, top services, ACSM survey

## Abstract

The physical activity, exercise and wellness sector is rapidly growing and seems to be an exciting field for business and professional development with great potential globally. The purpose of this observational and cross-sectional study was to determine the most popular health and fitness trends in Southern Europe for the first time, including data from Italy, Spain, Portugal, Greece and Cyprus, and to investigate any potential differences in this area compared to the Pan-European and global fitness trends for 2023. A national online survey was conducted in five Southern European countries, using the methodology of similar regional and worldwide surveys conducted by the American College of Sports Medicine since 2007. In total, a web-based questionnaire was sent to 19,887 professionals who worked in the Southern European physical activity, exercise and wellness sector. A total of 2645 responses were collected from five national surveys with an overall mean response rate of 13.3%. The ten most important fitness trends in Southern Europe for 2023 were personal training, licensure for fitness professionals, exercise is medicine, employing certified fitness professionals, functional fitness training, small group training, high-intensity interval training, fitness programs for older adults, post-rehabilitation classes and body weight training. The present findings are aligned with those reported for the European and worldwide fitness trends.

## 1. Introduction

The European health and fitness industry has experienced huge growth in the past 10 years, showing a significantly increased number of all industry stakeholders, such as entrepreneurs, employees, customers and businesses [1]. Although this particular service industry has currently passed through an economic crisis in the past decade [2] as well as an unbelievably difficult two-year period due to the coronavirus pandemic (COVID-19), recent evidence and popular trends have been documented, highlighting the merit of fitness services either digitally or on-site worldwide [3]. Small and medium-sized enterprises represent the vast majority of all businesses in the European Union (EU) [4], playing a key role in the economy while demonstrating a business field characterized by innovation, continuous development and great potential for the future [5]. However, the inactivity and obesity epidemics seem to be the most challenging global public health issues nowadays, affecting more than 50% of the adult population in the Western world [6],[7]. On the other side, regular exercise appears as a vital strategy for preventing, managing and treating several chronic diseases [8],[9].

The four largest (Italy, Spain, Portugal and Greece) and one of the smallest (Cyprus) Southern European countries selected for this study represent almost 30% of the EU population, showing a similar socioeconomic status compared to other EU members located in the North, West and East [10]. In these countries, the prevalence of the most common cardiometabolic health-related diseases is on the rise, creating significant concerns for the public health status and adversely affecting important physiological and psychological health markers compared to the mean of the EU countries [11]. Remarkably, several lifestyle-related health issues such as sedentarism, unhealthy weight, glucose intolerance, raised blood pressure and impaired blood lipid profile significantly impact the masses in Southern Europe [11],[12]. However, the latest available data just before the COVID-19 pandemic show promising insights into the annual revenue, market size, penetration and growth reported for all selected countries [13]. More specifically, Italy ranked fifth among all European fitness markets, demonstrating total revenue of EUR 2.3 billion, 5.5 million customers, 0.9% growth rate and 9.5% penetration rate [13]. Spain is the fourth largest national fitness market in Europe, with total revenue of EUR 2.4 billion, 5.5 million members in fitness clubs and growth and penetration rates of 3.3% and 13%, respectively [13]. Notably, Italy and Spain represent 16% of the total European market. On the other hand, Portugal and Greece are smaller fitness markets compared to Italy and Spain, and they are not included in the top 10 European national markets [1],[13]. Portugal and Greece display similar key financial figures with total revenues of EUR 0.26 billion and EUR 0.22 billion and penetration rates of 6.7% and 6.5%, respectively [13]–[15]. No data are available for the health and fitness market in Cyprus [16].

Since 2006, the American College of Sports Medicine (ACSM), as the leading organization on sports medicine and exercise science, has established a global annual survey on health and fitness trends, aiming to detect the most popular, valuable and safe exercise modes within the commercial, community, clinical and corporate health and fitness sectors [17]–[33]. Such a research approach may support customers to engage in positive exercise experiences while creating impactful prospects for exercise professionals, shaping novel business opportunities for gym operators in the health and fitness industry [33]. The statuses of health and fitness trends in Italy, Spain, Portugal, Greece and Cyprus have been studied once, six times, thrice, twice and once, respectively, investigating in depth the most attractive health and fitness trends in the past few years [34]–[44]. On the other side, the ACSM's worldwide survey collects data from different regions, providing international comparative analyses [45]–[49]. To date, several regional and national surveys replicating the ACSM's methodology have been published in order to distribute their findings and create awareness of the top health and fitness trends [34]–[44],[50]–[53].

This is the first research attempt to gather data from the largest Southern European countries and intends to compare the results found in the different countries of the same European region but also to compare them with those reported for Europe [49] and worldwide [33]. Although a Pan-European survey investigating this topic by collecting data from 40 countries is able to provide important evidence, such a study does not seem to be the optimal approach for providing reliable data for Italy, Spain, Portugal, Greece and Cyprus because of its varied sample size and the limited enrollment of participants from each selected country [51]. Thus, the primary aims of this survey were a) to detect the most important health and fitness trends in Southern Europe for 2023, b) to explore any potential differences in this field among Italy, Spain, Portugal, Greece and Cyprus and c) to compare the main outcomes with those reported for Europe and globally. Such a study may support the decision makers in the health and fitness industry in order to help all stakeholders support public health, which is substantially affected by obesity and inactivity in the Southern European region.

## 2. Materials and methods

### 2.1. Study design

An observational and cross-sectional study of health and fitness trends was conducted, using an online survey and a descriptive approach. The present cross-sectional study applied the same methodology with relevant surveys conducted by the ACSM, using similar criteria to those that have been commonly used in relevant national [34]–[44],[50]–[53], regional [45]–[50] and worldwide [17]–[33] surveys of fitness trends in the past 15 years. In this paper, data collected from five different national surveys are presented in order to compare the main findings not only among the five selected Southern European countries but also with those observed at European [49] and global levels [33]. In brief, the survey was developed to detect the trends (not fads) that are considered popular because of their positive influence on the physical activity, exercise and wellness sector while showing high attractiveness among key industry stakeholders based in all five selected Southern European countries, involving fitness services with a wide spectrum of clients. As such, a distinction between a “fad” and a “trend” according to the dictionary was included in the introduction of the survey to help participants recognize the difference between these two key terms [33].

### 2.2. Sample recruitment and inclusion criteria

Eligible participants included all adults aged 18 and older with any occupational role, experience and education level, work status and annual salary within the health and fitness industry. Databases of contacts of local universities (departments and schools of physical education, exercise or sports sciences) across all selected countries were primarily used to recruit participants for this survey. In addition, national associations of gym owners and national registries of exercise professionals in all involved countries were also used to collect data. The online survey was sent electronically to 19,887 individual contacts in total. All contacts were stakeholders in the physical activity, exercise and wellness industry based in Italy, Spain, Portugal, Greece and Cyprus under various occupational roles (e.g., employees, freelancers, students, educators, fitness club operators and managers). Likewise, the survey was posted on relevant web sites and social media (e.g., Facebook, Instagram, Twitter and LinkedIn) accounts of all involved parties.

### 2.3. Data collection tool

According to the ACSM's methodology, a technical experts group consisting of experienced practitioners and educators in the physical activity, exercise and wellness sector as well as academia was recruited to review a list of previously recognized trends as well as some new emerging trends [33]. Thus, a web-based questionnaire using an online survey platform (Google Forms, Survio or SurveyMonkey) was developed, including 40–50 related trends that were retrieved from several sources and from the personal experiences of some experts. A short explanation for each trend was provided to help participants become familiar with a basic description as previously reported [33]. The submitted responses on the potential trends were evaluated using a 10-point Likert scale ranging from 1 (least likely to be a trend) to 10 (most likely to be a trend) as previously described [33]. Demographic questions (e.g., gender, age, education, occupation, experience, work status, career choice and annual salary) were also included in the questionnaire that was developed to be completed in less than 15 minutes. The questionnaire was provided in local languages without any change from the original edition designed by the ACSM in English. The authors from each country were in charge of translation into local languages. Afterwards, native/bilingual English speakers with an academic background in the field of exercise science reviewed the draft, aiming to revise it where needed in order to be 100% correct compared to the original English version.

### 2.4. Recruitment and study period

The research was conducted electronically from May 2022 to July 2022 in Spain, Portugal and Greece and from September 2022 to November 2022 in Italy and Cyprus. No incentives (financial or material) were offered to attract participants to the survey in all countries. Several reminders were sent to all contacts via email during the study period. The online survey was filled out anonymously, and an informed consent letter was signed by all participants at the commencement of the electronic questionnaire. The first page of the online questionnaire included all required details regarding the research aims, confidentiality of information and the right to withdraw the participation in the study.

### 2.5. Data analysis

Given that the present survey was an explanatory study methodologically based on several similar studies widely conducted by the ACSM and associates [17]–[54], the information collected was analyzed using quantitative methods presented in the form of percentages of the respondents and the mean scores of the candidate trends, aiming to summarize the main findings of a large data sample and its measurements in a given moment. All descriptive analyses were performed using the IBM SPSS Statistics 25.0 software (IBM Corp., Armonk, NY, USA).

## 3. Results

In total, the five national online surveys collected 2645 responses, which represents a return rate of 13.3%. According to the demographic characteristics (Table 1), respondents (43% females and 57% males) with diverse backgrounds and experience levels participated in the study. Specifically, 44% of respondents had over 10 years of experience in the industry, and 15% had over 20 years of experience. Furthermore, 61% of respondents currently work as practitioners under various occupational roles, mostly as personal trainers (26%), while 74% of participants held a bachelor's degree in exercise science or a related field. Full-time work and number one career choice were stated by 38% and 73%, respectively. Lastly, 71% of participants reported an annual salary lower than EUR 20,000. All candidate trends were ranked from highest (most popular trend) to lowest (least popular trend) mean score and are illustrated in Table 2. A comparison of the top 20 fitness trends among Italy, Spain, Portugal, Greece, Cyprus, Europe and the world is shown in Table 3. Trends were also categorized in the following six groups, as articulated elsewhere [54]: trends related to i) fitness professionals, ii) fitness activities, iii) training modalities, iv) programs oriented to specific populations, v) technology and iv) health. Table 4 presents a grouped approach of the comparative analysis of the top 20 fitness trends in Italy, Spain, Portugal, Greece, Cyprus, Europe and the world.

**Figure 1.**
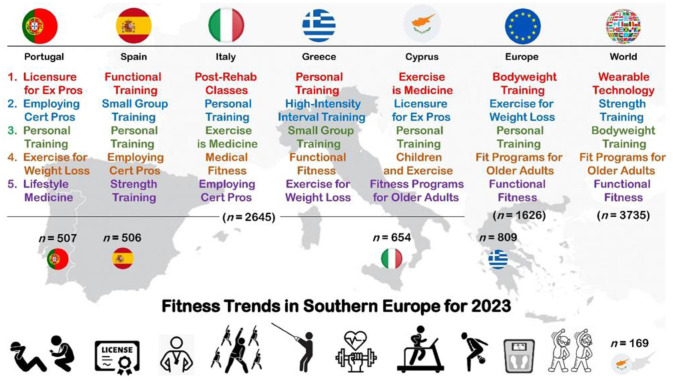
Top five health and fitness trends in Portugal, Spain, Italy, Greece, Cyprus, Europe and worldwide.

In brief, personal training was the only common trend included in the top three selections in all Southern European countries for 2023. On average, personal training, employing certified fitness professionals, licensure for fitness professionals, exercise is medicine, functional fitness training, small group training and exercise for weight loss were widely selected as some of the seven most popular trends in the Southern European region. More specifically, the two trends related to fitness professionals (employing certified fitness professionals and licensure for fitness professionals) included in the survey were present on the list of top 20 fitness trends in all selected Southern European countries. Similarly, five trends related to fitness activities (outdoor activities, high-intensity interval training, functional fitness training, strength training with free weights and body weight training), four trends related to fitness modalities (personal training, small group training, health/wellness coaching and group training), three trends related to programs oriented to specific populations (exercise for weight loss, fitness programs for older adults and children and exercise) and three trends related to health (post-rehabilitation classes, exercise is medicine and lifestyle medicine) were ranked among the top 20 most attractive options regionally. Interestingly, some trends related to fitness activities were popular in specific countries, such as circuit training (Greece and Cyprus), core training (Spain and Greece) and Pilates (Portugal, Greece and Cyprus). In addition, a large majority of trends related to technology were not included in top 20, and only outcome measurements and wearable technology were present on the top 20 list, but not in all countries. Comparing the top 20 Southern European fitness trends with the Pan-European and worldwide ones published by the ACSM for 2023, there is one trend (boutique fitness studios) that is included only in the European list, and there are two trends (home exercise gyms and yoga) included only in the global list. Figure 1 illustrates the top five health and fitness trends in each Southern European country included in the present study in comparison to the European [49] and worldwide [33] ACSM surveys for 2023.

**Table 1.** Demographics of the survey respondents in Italy, Spain, Portugal, Greece and Cyprus.

		Italy (*n* = 654)		Spain (*n* = 506)		Portugal (*n* = 507)		Greece (*n* = 809)		Cyprus (*n* = 169)	
		N	%	N	%	N	%	N	%	N	%
*Gender*				
	Female	242	37.0	229	31.2	261	51.5	357	44.1	54	32.0
	Male	412	63.0	348	68.8	246	48.5	452	55.9	115	68.0
*Age (years)*				
	18–21	252	38.5	9	1.8	56	11.0	111	13.7	21	12.4
	22–34	334	51.1	146	28.9	215	42.4	320	39.6	92	54.5
	35–44	30	4.6	174	34.4	168	33.2	244	30.2	32	18.9
	45–54	16	2.5	133	26.5	45	8.9	102	12.6	24	14.2
	55–64	20	3.0	35	6.7	19	3.7	27	3.3	0	0.0
	65+	2	0.3	9	1.8	4	0.8	4	0.5	0	0.0
*Education*				
	Undergraduate student	0	0.0	N/A	N/A	0	0.0	0	0.0	33	19.5
	Vocational education and training	222	33.9	N/A	N/A	121	23.9	123	15.2	51	30.2
	Bachelor's degree	312	47.7	N/A	N/A	279	55.0	343	42.4	66	39.1
	Master's degree	83	12.7	N/A	N/A	83	16.4	308	38.1	9	5.3
	Doctoral degree	37	5.7	N/A	N/A	24	4.7	35	4.3	10	5.9
*Occupation*				
	Gym Owner/Operator	14	2.1	102	20.2	120	23.7	119	14.7	22	13.0
	Gym Manager	8	1.2	120	23.8	0	0.0	47	5.8	11	6.5
	Fitness Instructor	13	2.0	28	5.5	235	46.4	92	11.4	0	0.0
	Group Fitness Instructor	18	2.8	113	22.3	245	48.3	72	8.9	19	11.2
	Personal Trainer	59	9.0	105	20.8	295	58.2	176	21.8	41	24.3
	Pilates Instructor	15	2.3	N/A^1^	N/A	N/A^1^	N/A	46	5.7	7	4.1
	Yoga Instructor	12	1.8	N/A^1^	N/A	N/A^1^	N/A	39	4.8	0	0.0
	CrossFit-based Training Instructor	0	0.0	N/A^1^	N/A	60	11.8	37	4.5	0	0.0
	Undergraduate Student	397	60.7	4	0.8	41	8.1	97	12.0	35	20.7
	Graduate Student	10	1.5	5	1.0	91	17.9	17	2.1	1	0.6
	Exercise Physiologist	3	0.5	0	0.0	0	0.0	15	1.9	1	0.6
	Clinical Exercise Physiologist	2	0.3	0	0.0	0	0.0	176	21.8	2	1.2
	Physical Education Teacher	24	3.7	10	2.0	0	0.0	19	2.3	17	10.1
	Sports Coach	45	6.9	0	0.0	0	0.0	0	0.0	0	0.0
	Vocational Educator/Tutor	2	0.3	0	0.0	0	0.0	4	0.5	5	3.0
	University Professor	30	4.6	0	0.0	97	19.1	29	3.6	8	4.7
	Medical Professional	2	0.3	4	0.8	28	5.5	0	0.0	0	0.0
	Other	0	0.0	25	4.9	30	5.9	0	0.0	0	0.0
*Experience (years)*	
	0–5	452	69.1	66	13.1	151	29.7	175	21.6	67	39.6
	6–10	91	13.9	122	24.1	93	18.3	274	33.9	30	17.8
	10–20	83	12.7	173	34.2	184	36.3	228	28.2	52	30.8
	20+	28	4.3	145	28.6	79	15.7	132	16.3	20	11.8
*Work status*								
	Full-time	131	20.0	332	65.6	208	41.0	330	48.7	0	0.0
	Part-time	523	80.0	136	26.9	299	59.0	179	32.1	62	36.7
	Hourly basis	0	0.0	38	7.5	0	0.0	33	19.2	107	63.3
*Career choice*				
	First job	542	82.9	408	80.6	395	77.9	448	55.4	131	77.5
	Second job	83	12.7	41	8.1	112	22.1	273	33.7	25	14.8
	Third job	29	4.4	57	11.3	0	0.0	88	10.9	13	7.7
*Annual salary (€)*				
	<20,000	580	88.7	252	49.8	254	50.1	695	85.9	106	62.7
	20,000–29,999	27	4.1	117	23.1	28	5.5	76	9.4	34	20.1
	30,000–39,999	13	2.0	69	13.6	7	1.4	26	3.2	13	7.7
	40,000–49,999	9	1.4	34	6.8	4	0.8	8	1.0	6	3.5
	50,000–59,999	11	1.7	34	6.7	5	1.2	4	0.5	2	1.2
	60,000–69,999	3	0.5	0	0.0	0	0.0	0	0.0	3	1.8
	70,000–79,999	4	0.6	0	0.0	0	0.0	0	0.0	1	0.6
	80,000–89,999	5	0.7	0	0.0	0	0.0	0	0.0	1	0.6
	90,000–99,999	0	0.0	0	0.0	0	0.0	0	0.0	1	0.6
	>100,000	2	0.3	0	0.0	0	0.0	0	0.0	2	1.2

*Note: ^1^included in group fitness instructors; N/A – not available.

**Table 2.** Comprehensive ranking of fitness trends in Italy, Spain, Portugal, Greece and Cyprus for 2023.

#	Italy		Spain		Portugal		Greece		Cyprus	
	Trend	Score	Trend	Score	Trend	Score	Trend	Score	Trend	Score
1	Post-Rehabilitation Classes	9.086	Functional Fitness Training	8.360	Licensure for Fitness Professionals	8.840	Personal Training	8.320	Exercise is Medicine	9.355
2	Personal Training	8.931	Small Group Training	8.261	Employing Certified Fitness Professionals	8.790	High-Intensity Interval Training	8.110	Licensure for Fitness Professionals	9.231
3	Exercise is Medicine	8.916	Personal Training	8.249	Personal Training	8.440	Small Group Training	8.060	Personal Training	8.964
4	Clinical Integration / Medical Fitness	8.867	Employing Certified Fitness Professionals	8.229	Exercise for Weight Loss	8.310	Functional Fitness Training	8.020	Children and Exercise	8.947
5	Employing Certified Fitness Professionals	8.723	Strength Training (Free-Weights)	8.065	Lifestyle Medicine	8.120	Exercise for Weight Loss	7.860	Fitness Programs for Older Adults	8.882
6	Long-term Youth Development	8.722	Exercise and Weight Loss	8.061	Health/Well-Being Coaching	8.110	High-Intensity Functional Training	7.750	Functional Fitness Training	8.840
7	Children and Exercise	8.661	Fitness Programs for Older Adults	7.850	Exercise is Medicine	8.109	Body Weight Training	7.680	Employing Certified Fitness Professionals	8.817
8	Fitness Programs for Older Adults	8.630	Multidisciplinary Work Teams^1^	7.846	Strength Training (Free Weights)	8.020	Fitness Programs for Older Adults	7.550	Health/Wellness Coaching	8.775
9	Outdoor Activities	8.560	Licensure for Fitness Professionals	7.838	Outcome Measurements	7.950	Exercise is Medicine	7.520	Post Rehabilitation Classes	8.751
10	Worksite Health Promotion Programs	8.555	Outdoor Activities	7.816	Outdoor Activities	7.880	Group Training	7.540	Small Group Training	8.716
11	Licensure for Fitness Professionals	8.540	High-Intensity Interval Training	7.739	Functional Fitness Training	7.840	Pilates	7.390	Circuit Training	8.669
12	Exercise for Weight Loss	8.539	Post Rehabilitation Classes	7.735	Body Weight Training	7.760	Boutique Fitness Studios	7.260	Body Weight Training	8.633
13	Pre- and Post-natal Fitness	8.538	Fitness and Nutrition ^1^	7.733	Fitness Programs for Older Adults	7.750	Strength Training (Free Weights)	7.200	Group Training	8.627
14	Lifestyle Medicine	8.514	Body Weight Training	7.727	Body-Mind Movement	7.700	Outdoor Activities	7.140	Exercise for Weight Loss	8.604
15	Health/Well-Being Coaching	8.402	Injury Prevention / Functional Rehab ^1^	7.674	High-Intensity Interval Training	7.680	Circuit Training	7.130	Pilates	8.598
16	Outcome Measurements	8.361	Group Training	7.536	Post Rehabilitation Classes	7.650	Licensure for Fitness Professionals	7.120	Strength Training (Free Weights)	8.550
17	Functional Fitness Training	8.353	Outcome Measurement	7.530	Pilates	7.640	Health/Wellness Coaching	7.110	High-Intensity Interval Training	8.544
18	Balance and Stabilization Training	8.309	Core Training	7.526	Wearable Technology	7.630	Employing Certified Fitness Professionals	7.100	Core Training	8.515
19	Strength Training (Free Weights)	8.292	Mobile Exercise Apps	7.474	High-Intensity Functional Training	7.610	Wearable Technology	7.090	Clinical Integration / Medical Fitness	8.426
20	Stretching Training	8.208	Exercise is Medicine	7.380	Group Training	7.600	Core Training	7.060	Outcome Measurements	8.373
21	Core Training	8.150	Wearable technology	7.373	Clinical Integration / Medical Fitness	7.580	Home Exercise Gyms	6.970	Lifestyle Medicine	8.331
22	Body Weight Training	8.142	Seeking New Market Niches	7.269	Circuit Training	7.520	Post Rehabilitation Classes	6.940	High-Intensity Functional Training	8.260
23	High-Intensity Functional Training	8.095	Boutique Fitness Studios	7.251	Core Training	7.510	Clinical Integration / Medical Fitness	6.900	Outdoor Activities	8.254
24	High-Intensity Interval Training	8.075	Children with Obesity and Exercise ^1^	7.213	Pre- and Post-natal Fitness	7.490	Yoga	6.800	Walking/Running/ Jogging/Cycling Clubs	8.249
25	Wearable Technology	8.006	Circuit Training	7.190	Small Group Training	7.480	Walking/Running/ Jogging/Cycling Clubs	6.760	Resistance Band Training	8.195
26	Circuit Training	7.969	Worksite Health Promotion Programs	7.174	Children and Exercise	7.340	Online Live/On-Demand Exercise Classes	6.720	Aquatic Exercise	8.154
27	Aquatic Exercise	7.812	Postural Correction	7.158	Mobile Exercise Apps	7.280	Online Personal Training	6.660	Stretching Training	8.136
28	Worker Incentive Program	7.797	Lifestyle Medicine	7.144	CrossFit-based Training	7.200	Pre- and Post-natal Fitness	6.570	Boutique Fitness Studios	8.130
29	Group Training	7.615	Physician Referrals to Fitness Programs	7.065	Online Personal Training	7.080	Children and Exercise	6.380	Balance and Stabilization Training	8.118
30	Small Group Training	7.567	Pre- and Post-natal Fitness	6.919	Worksite Health Promotion Programs	7.070	Low-cost and Budget Gyms	6.290	Wearable Technology	8.071
31	Resistance Band Training	7.433	Walking/Running/ Jogging/Cycling Clubs	6.883	Long-term Youth Development	7.060	Mobility/Myofascial Devices and Recovery	6.190	Worker Incentive Programs	8.059
32	Plyometric Training	7.316	Pilates	6.840	Worker Incentive Programs	6.990	Stretching Training	6.080	Pilates	8.041
33	Walking/Running/ Jogging/Cycling Clubs	7.277	Yoga	6.836	Low-cost and Budget Gyms	6.920	Mobile Exercise Apps	6.060	Worksite Health Promotion Programs	8.012
34	Pilates	7.170	Clinical Integration / Medical Fitness	6.828	Walking/Running/ Jogging/Cycling Clubs	6.900	Balance and Stabilization Training	6.030	Pre- and Post-Natal Fitness	7.982
35	Yoga	7.170	Exercise for Children	6.798	Resistance Band Training	6.890	Worksite Health Promotion Programs	6.010	Plyometric Training	7.793
36	Medicine Ball Training	7.008	Health/Wellness Coaching	6.749	Home Exercise Gyms	6.770	Lifestyle Medicine	5.920	Boxing, Kickboxing & Mixed Martial Arts	7.769
37	Home Exercise Gyms	6.888	Online Personal Training	6.690	Balance and Stabilization Training	6.760	Worker Incentive Programs	5.170	Yoga	7.556
38	Low-cost and Budget Gyms	6.688	Balance and Stabilization Training	6.581	Stretching Training	6.730	Outcome Measurements	5.150	Mobility/Myofascial Devices and Recovery	7.491
39	Mobile Exercise Apps	6.520	Mobility/Myofascial Devices and Recovery	6.571	Yoga	6.720	Long-term Youth Development	5.110	Dance-based Workouts	7.172
40	Online Personal Training	6.416	Home Exercise Gyms	6.561	Eco Gyms	6.640	Dance-based Workouts	5.080	Mind-Body Movement	7.130
41	Online Live and On-Demand Exercise Classes	6.153	Online Live and On-Demand Exercise Classes	6.474	Mobility/Myofascial Devices and Recovery	6.630	Virtual Reality Exercise Training	5.050	Home Exercise Gyms	7.095
42	Electrical Muscle Stimulation Training	6.107	Aquatic Exercise	6.326	Boutique Fitness Studios	6.620	Electrical Muscle Stimulation Training	5.020	Boot Camp-Style	6.852
43	Dance-based Workouts	6.041	Exercise Post-COVID Recuperation Programs	6.294	Fitness Influencers	6.610	Aquatic Exercise	4.780	Low-cost and Budget Gyms	6.479
44	Virtual Reality Exercise Training	5.771	Resistance Band Training	6.166	Aquatic Exercise	6.350	Mind-Body Movement	4.770	Online Live and On-Demand Exercise Classes	6.444
45	Mind-Body Movement	5.725	Dance-based Workouts	6.117	Dance-based Workouts	6.180			Mobile Exercise Apps	6.361
46			Plyometric Training	5.960	Plyometric Training	5.980			Online Personal Training	6.296
47			Virtual Reality Exercise Training	5.700	Online Live and On-Demand Exercise Classes	5.820			Online Training	6.006
48					Medicine Ball Training	5.640			Virtual Reality Training	4.675
49					Virtual Reality Exercise Training	5.360			Electrical Muscle Stimulation Training	4.402
50					Electrical Muscle Stimulation Training	5.300				

*Note: ^1^appearance only in Spain; Scores are expressed as mean.

**Table 3.** Comparative analysis of top 20 fitness trends among Italy, Spain, Portugal, Greece, Cyprus, Europe and the world.

#	Italy	Spain	Portugal	Greece	Cyprus	Europe [49]	World [33]
1	Post-Rehabilitation Classes	Functional Fitness Training	Licensure for Fitness Professionals	Personal Training	Exercise is Medicine	Body Weight Training	Wearable Technology
2	Personal Training	Small Group Training	Employing Certified Fitness Professionals	High-Intensity Interval Training	Licensure for Fitness Professionals	Exercise for Weight Loss	Strength Training (Free Weights)
3	Exercise is Medicine	Personal Training	Personal Training	Small Group Training	Personal Training	Personal Training	Body Weight Training
4	Clinical Integration /Medical Fitness	Employing Certified Fitness Professionals	Exercise for Weight Loss	Functional Fitness Training	Children and Exercise	Fitness Programs for Older Adults	Fitness Programs for Older Adults
5	Employing Certified Fitness Professionals	Strength Training (Free-Weights)	Lifestyle Medicine	Exercise for Weight Loss	Fitness Programs for Older Adults	Functional Fitness Training	Functional Fitness Training
6	Long-term Youth Development	Exercise and Weight Loss	Health/Well-Being Coaching	High-Intensity Functional Training	Functional Fitness Training	High-Intensity Interval Training	Outdoor Activities
7	Children and Exercise	Fitness Programs for Older Adults	Exercise is Medicine	Body Weight Training	Employing Certified Fitness Professionals	Boutique Fitness Studios	High-Intensity Interval Training
8	Fitness Programs for Older Adults	Multidisciplinary Work Teams ^1^	Strength Training (Free Weights)	Fitness Programs for Older Adults	Health/Wellness Coaching	Circuit Training	Exercise for Weight Loss
9	Outdoor Activities	Licensure for Fitness Professionals	Outcome Measurements	Exercise is Medicine	Post-Rehabilitation Classes	Exercise is Medicine	Employing Certified Fitness Professionals
10	Worksite Health Promotion Programs	Outdoor Activities	Outdoor Activities	Group Training	Small Group Training	Employing Certified Fitness Professionals	Personal Training
11	Licensure for Fitness Professionals	High-Intensity Interval Training	Functional Fitness Training	Pilates	Circuit Training	Strength Training (Free Weights)	Core Training
12	Exercise for Weight Loss	Post-Rehabilitation Classes	Body Weight Training	Boutique Fitness Studios	Body Weight Training	Wearable Technology	Circuit Training
13	Pre- and Post-natal Fitness	Fitness & Nutrition ^1^	Fitness Programs for Older Adults	Strength Training (Free Weights)	Group Training	High-Intensity Functional Training	Home Exercise Gyms
14	Lifestyle Medicine	Body Weight Training	Body-Mind Movement	Outdoor Activities	Exercise for Weight Loss	Outdoor Activities	Group Training
15	Health/Well-Being Coaching	Injury Prevention / Functional Rehab ^1^	High-Intensity Interval Training	Circuit Training	Pilates	Clinical Integration / Medical Fitness	Exercise is Medicine
16	Outcome Measurements	Group Training	Post-Rehabilitation Classes	Licensure for Fitness Professionals	Strength Training (Free Weights)	Small Group Training	Lifestyle Medicine
17	Functional Fitness Training	Outcome Measurement	Pilates	Health/Wellness Coaching	High-Intensity Interval Training	Children and Exercise	Yoga
18	Balance & Stabilization Training	Core Training	Wearable Technology	Employing Certified Fitness Professionals	Core Training	Licensure for Fitness Professionals	Licensure for Fitness Professionals
19	Stretching Training	Mobile Exercise Apps	High-Intensity Functional Training	Wearable Technology	Clinical Integration / Medical Fitness	Pilates	Health/Well-Being Coaching
20	Strength Training (Free Weights)	Exercise is Medicine	Group Training	Core Training	Lifestyle Medicine	Group Training	Mobile Exercise Apps

*Note: ^1^appearance only in Spain; Scores are expressed as mean.

**Table 4.** A grouped comparative analysis of top 20 fitness trends in Italy, Spain, Portugal, Greece, Cyprus, Europe and the world.

#	Italy	#	Spain	#	Portugal	#	Greece	#	Cyprus	#	Europe [49]	#	World [33]
*Trends related to fitness professionals:*					
5	Employing Certified Fitness Professionals	4	Employing Certified Fitness Professionals	1	Licensure for Fitness Professionals	18	Employing Certified Fitness Professionals	2	Licensure for Fitness Professionals	10	Employing Certified Fitness Professionals	9	Employing Certified Fitness Professionals
11	Licensure for Fitness Professionals	9	Licensure for Fitness Professionals	2	Employing Certified Fitness Professionals	16	Licensure for Fitness Professionals	7	Employing Certified Fitness Professionals	18	Licensure for Fitness Professionals	18	Licensure for Fitness Professionals
*Trends related to fitness activities:*						
9	Outdoor Activities	1	Functional Fitness Training	8	Strength Training (Free Weights)	2	High-Intensity Interval Training	6	Functional Fitness Training	1	Body Weight Training	2	Strength Training (Free Weights)
17	Functional Fitness Training	5	Strength Training (Free Weights)	10	Outdoor Activities	4	Functional Fitness Training	11	Circuit Training	5	Functional Fitness Training	3	Body Weight Training
18	Balance and Stabilization Training	10	Outdoor Activities	11	Functional Fitness Training	7	Body Weight Training	12	Body Weight Training	6	High-Intensity Interval Training	5	Functional Fitness Training
19	Stretching Training	11	High-Intensity Interval Training	12	Body Weight Training	15	Circuit Training	15	Pilates	7	Boutique Fitness Studios	6	Outdoor Activities
20	Strength Training (Free Weights)	14	Body Weight Training	14	Mind-Body Movement	19	Strength Training (Free Weights)	16	Strength Training (Free Weights)	8	Circuit Training	7	High-Intensity Interval Training
		18	Core Training	15	High-Intensity Interval Training	14	Outdoor Activities	17	High-Intensity Interval Training	11	Strength Training (Free Weights)	11	Core Training
				17	Pilates	6	High-Intensity Functional Training	18	Core Training	13	High-Intensity Functional Training	12	Circuit Training
				19	High-Intensity Functional Training	11	Pilates			14	Outdoor Activities	13	Home Exercise Gyms
						20	Core Training			19	Pilates	17	Yoga
						12	Boutique Fitness Studios						
*Trends related to training modalities:*							
21	Personal Training	2	Small Group Training	3	Personal Training	1	Personal Training	3	Personal Training	3	Personal Training	10	Personal Training
5	Health/Wellness Coaching	3	Personal Training	6	Health/Wellness Coaching	3	Small Group Training	8	Health/Wellness Coaching	16	Small Group Training	14	Group Training
		16	Group Training	20	Group Training	10	Group Training	10	Small Group Training	20	Group Training	19	Health/Wellness Coaching
						17	Health/Wellness Coaching	13	Group Training				
*Trends related to programs oriented to specific populations:*							
6	Long-term Youth Development	6	Exercise for Weight Loss	4	Exercise for Weight Loss	5	Exercise for Weight Loss	4	Children and Exercise	2	Exercise for Weight Loss	4	Fitness Programs for Older Adults
7	Children and Exercise	7	Fitness Programs for Older Adults	12	Fitness Programs for Older Adults	8	Fitness Programs for Older Adults	5	Fitness Programs for Older Adults	4	Fitness Programs for Older Adults	8	Exercise for Weight Loss
8	Fitness Programs for Older Adults							14	Exercise for Weight Loss	17	Children and Exercise		
12	Exercise for Weight Loss												
13	Pre- and Post-natal Fitness												
*Trends related to technology:*							
16	Outcome Measurements	17	Outcome Measurements	9	Outcome Measurements	19	Wearable Technology			12	Wearable Technology	1	Wearable Technology
		19	Mobile Exercise Apps	18	Wearable Technology							20	Mobile Exercise Apps
*Trends related to health:*						
1	Post-Rehabilitation Classes	8	Multidisciplinary Work Teams	5	Lifestyle Medicine	9	Exercise is Medicine	1	Exercise is Medicine	9	Exercise is Medicine	15	Exercise is Medicine
3	Exercise is Medicine	12	Post-Rehabilitation Classes	7	Exercise is Medicine			9	Post-Rehabilitation Classes	15	Clinical Integration/Medical Fitness	16	Lifestyle Medicine
4	Clinical Integration /Medical Fitness	13	Fitness & Nutrition	16	Post-Rehabilitation Classes			19	Clinical Integration /Medical Fitness				
14	Lifestyle Medicine	15	Injury Prevention					20	Lifestyle Medicine				
		20	Exercise is Medicine										

## 4. Discussion

### 4.1. Main results in brief

An online survey of health and fitness trends in Southern Europe, including Italy, Spain, Portugal, Greece and Cyprus, was carried out for the first time, aiming to help all industry stakeholders detect the current state of the health and fitness trends linked to specific services and programs. Moreover, it may help employers, health club managers, fitness professionals and educators to enhance customer engagement and experience through evidence-based practices and attractive strategies in this vibrant sector. In Southern Europe, personal training, licensure for fitness professionals, exercise is medicine, employing certified fitness professionals, functional fitness training, small group training, high-intensity interval training, fitness programs for older adults, post-rehabilitation classes and body weight training were identified as the top 10 trends for 2023. Interestingly, almost 50% of top 20 selections are trends related to fitness activities associated with various exercise types and settings. Trends related to fitness modalities and health were the second and third most attractive categories of trends, respectively. In contrast, technology-oriented trends showed low popularity, while specific population-oriented trends demonstrated significant potential among selected countries in the Southern European region. Importantly, the majority of mind-body fitness modalities such as yoga, tai chi and mind-body movement were not ranked in top 20, indicating low attractiveness in Southern Europe. However, Pilates showed a greater popularity than other mind-body fitness activities, since it was included in the top 20 list in three out of five selected countries in the region.

### 4.2. What is most popular?

The results of the first-ever Southern European survey of fitness trends using the ACSM's methodology [33] show several common findings with other recently published observational studies investigating fitness trends in several countries [34]–[44],[50]–[53] and regions [45]–[50]. On average, personal training was ranked #1 in the present study, which is an outcome that agrees with the findings reported in Europe [49] but not the world for 2023 [33]. Moreover, this particular trend appears to be popular globally as the tenth most attractive fitness trend [33] while it was ranked #8 in the USA, #1 in Brazil, #3 in Mexico, #3 in Europe, #7 in Australia and #17 in China [49]. Personal training sessions seem to be remarkably popular among Europeans, and therefore the big majority (75%) of fitness ser-vices offered in health clubs and boutique fitness studios are primarily focused on the one-on-one exercise setting [55]. Furthermore, the occupation of personal trainer has been reported as the second most promising profession among European exercise professionals [56],[57]. The present results may underline the current state of the health and fitness sector, which seems to be linked to client-centered services, aiming to improve customer engagement, satisfaction and loyalty through individualized exercise experiences [58].

### 4.3. Fitness professionals: The frontline players

**Figure 2.**
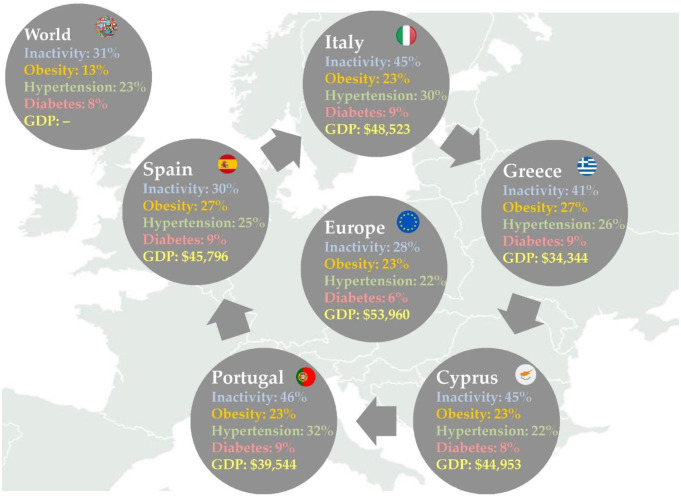
Health (noncommunicable diseases) and socioeconomic country profiles (*GDP – gross domestic product* [6],[7],[10],[11]).

Trends related to fitness professionals such as licensure for fitness professionals and employing certified fitness professionals seem substantially popular in the Southern European region, since both were reported as some of the top 20 trends in all five countries included in the present survey for 2023. It is of note that these particular trends have recently increased their popularity not only in Southern Europe but also in numerous other countries and regions globally [32],[33],[47]–[49]. The high demand for qualified practitioners is systematically growing because of the rapidly increasing prevalence of populations affected by several lifestyle-related cardiometabolic health diseases that negatively impact physical and mental health as well as life expectancy at the global level [7],[11]. The present results highlight that licensed, certified and well-equipped exercise professionals should be able to work on the front line against the inactivity epidemic by offering client-centered services based on good practices. This is an important finding considering that a significantly high percentage of the adult population in all five countries involved in this study are not apparently healthy individuals (Figure 2). More importantly, such populations belong to underserved markets [59], showing that personalized services through adapted fitness programs delivered by qualified training staff aiming to provide an inclusive environment appear to be a high priority among customers, employees and entrepreneurs in the health and fitness industry [60]. Interestingly, the occupational role of personal trainer has been selected as one of the most promising professions among practitioners in the European health and fitness industry [56],[57]. Such observations underline the key role of fitness professionals in public health [61], working as members of a multidisciplinary healthcare team in collaboration with physicians and allied healthcare practitioners in both gym and clinical settings [62],[63]. Examining the main results of the present cross-sectional study, it is important to indicate that there may be an interaction among trends related to exercise professionals and those related to health as well as some particular training modalities (e.g., personal training, small group training, health/wellness coaching and group training), since all of them are included in top 20 fitness trends among all five Southern European countries. However, this is an outcome that cannot be explained thoroughly here, and therefore further research is needed in the future in this area. Moreover, the first-ever Southern European survey of health and fitness trends may underline the rationale for regulation and licensing requirements for practitioners in the physical activity, exercise and wellness sector as applied by other allied healthcare professions (e.g., physical therapy, dietetics, chiropractic, osteopathic and nursing) globally, aiming to ensure higher standards for practitioners and protect consumers in the health and fitness sector. Lastly, given that fitness professionals based in Spain and Portugal demonstrated moderate to high levels of job satisfaction [64],[65] and quality of life [66], it seems that the physical activity, exercise and wellness industry is a vibrant sector full of professional opportunities and a pleasant work environment.

### 4.4. What is trending in fitness activities?

Functional fitness training, body weight training, high-intensity interval training (HIIT), strength training with free weights and outdoor activities are popular trends in all selected Southern European countries. Circuit training, Pilates and core training were popular mostly in Greece and Cyprus and somewhat popular in Spain and Portugal but not in Italy. These observations are aligned with those reported in Europe [49] and worldwide [33] for 2023. Their attractiveness may be gradually growing in the worldwide health and fitness industry since these particular trends are mainly linked to first-line services in boutique fitness studios, which appear to be an emerging type of fitness facility in Europe and globally [3],[67]. It is noteworthy that this particular workplace has been reported as one of the most promising gym settings among fitness professionals based in Europe [57]. However, boutique fitness studios are not currently popular in Southern Europe, since only in Greece was this option included in the top 20 fitness trends for 2023.

Considering that the physical activity, exercise and leisure sector has been significantly impacted by the COVID-19 pandemic in the past two years [1],[3] and a long-lasting socioeconomic crisis in the past decade [68], especially in Southern European countries that demonstrate lower socioeconomic status than those in Northern and Central Europe [69], the personal training space seems to be recovering according to the present results. In addition, boutique fitness studios appear as the next generation of gym facilities, aiming to enhance customer engagement by offering targeted training programs and services [58],[67]. On the other hand, conventional health clubs with large facilities may be still attractive in Southern Europe, since traditional fitness activities such as strength training with free weights and group training are reported as popular in all five countries in this region for 2023. It is noteworthy that group training classes delivering HIIT, functional fitness and bodyweight training classes have been reported as some of the principal fitness services in the European health and fitness sector [70]. Likewise, resistance training has been widely established as an essential component of every physical exercise program for health, performance and well-being [71]. However, outdoor activities gained a lot of popularity during the COVID-19 pandemic due to fitness club restrictions worldwide [32],[48]. This seems to be a trend that has retained its popularity even after that period, since it has been reported as one of the top 20 trends in Italy, Spain, Portugal and Greece, but not in Cyprus, for 2023. The currently increasing attractiveness of outdoor activities may be also supported by the fact that the running movement is systematically growing not only in Southern Europe but also globally [72].

In general, gym services related to group fitness and muscle-strengthening programs appear to be significantly popular to customers in some Southern European markets [2],[14],[15]. In the attempt to explain the attractiveness of the top trends related to fitness activities from a scientific perspective, the existing body of evidence shows that HIIT-based programs may be a user-friendly, effective, time-efficient and pleasant exercise option for the masses seeking to improve several physical and mental health markers in both group and small group training settings [73],[74]. Furthermore, recent evidence indicates that training programs integrating some of the most popular fitness activities (e.g., HIIT, functional fitness and body weight training) as a hybrid-type session into real-world gym conditions provide beneficial changes in several psychophysiological indicators [75] among people representing the big majority of the adult population in the Western world [11]. Interestingly, people with an unhealthy weight are also underlined in this study, since exercise for weight loss was included in the top 20 trends in all five Southern European countries for 2023.

Another important outcome from the Southern European survey that should be taken into consideration is the low ranking of trends related to mind-body fitness such as yoga and tai chi. These particular trends were absent from the top 20 among all five countries included in this study. This is a result ultimately in line with findings recently reported in other national and regional surveys [47]–[50]. Such alternative fitness activities may need a more user-friendly approach to regularly engage the masses in these meditative exercise types that require greater functional capacity and less movement dysfunction than other training modes [76]. Instead, Pilates may be more inclusive compared to other mind-body fitness modalities, showing higher popularity in the Southern European region for 2023 [77],[78]. However, Pilates demonstrates low attractiveness in other countries and regions [32]–[38],[47]–[50],[52],[53],[79], and this is an observation that may need additional investigation in the next years.

### 4.5. Which setting is most popular? Private, semi-private or group training?

Private and semi-private training settings are shown as more popular than group training settings among fitness industry stakeholders in Southern Europe for 2023. This fact underlines the importance of delivering personal and small group training sessions in a gym environment, aiming to offer more customized services and promoting engaging exercise experiences. According to the present study, on average, personal training has been selected as the most attractive trend. Small group training, as well as group training, also appear as popular trends in all Southern European countries involved in this survey. However, client-centered services through personal training sessions may experience higher demand than other training modalities among customers in the Southern European health and fitness industry, and this finding corroborates recently published data regarding the popularity of personal training in Europe [47]–[50]. Private training sessions may ensure a supervised environment that makes a substantial difference compared to other popular training modalities, since such a setting promotes an engaging exercise experience. This fact may be one of the greatest advantages of personal training compared to other trends related to fitness activities and modalities. An inclusive environment is likely to be provided by personalized training sessions, which seems to be critical for increasing customer engagement in the physical activity, exercise and wellness industry [58].

On the other side, group training involves exercise professionals instructing people through in-person group classes involving more than five participants. Group training classes are primarily designed for clients of all ages and all fitness levels, applying a variety of equipment to deliver several types of classes, from aerobic-based and indoor cycling classes to dance-based and step classes. Group training had been significantly impacted by the coronavirus pandemic in 2020–2022, and thus it was not listed high on the top 20 fitness trends worldwide [32],[47],[48]. Moreover, dance- and aquatic-based training programs demonstrate limited applicability in all five Southern European countries since both were ranked very low among all candidate fitness trends in this region. Lastly, the presence of health/wellness coaching, as a trend related to training modalities, among the top 20 trends in the Southern European region shows a potential interaction between this particular trend and various health-related trends. Such a finding highlights the important role of regular exercise in physical and mental health, indicating a new landscape in the fitness industry regionally.

### 4.6. What is going on with underserved markets?

In this Southern European survey, four out of the top 20 selections are related to health: post-rehabilitation classes, exercise is medicine, lifestyle medicine and clinical integration / medical fitness. Likewise, another four trends related to programs focused on specific populations, children and exercise, fitness programs for older adults, exercise for weight loss and pre- and post-natal fitness, were ranked in the top 20. These results indicate the potential of these trends for all fitness industry stakeholders, since various lifestyle-related cardiometabolic health issues currently demonstrate an increasing prevalence in Southern Europe. According to the World Health Organization, Italy, Spain, Portugal, Greece and Cyprus demonstrate similar country health profiles (Figure 2) [11], which may support the present findings showing that public health needs an enhancement by the physical activity, exercise and well-being sector at the national level. Interestingly, similar results can be found in other countries and regions, showing that various underserved markets may be under the microscope of the health and fitness sector. Such an observation shows that both businesses and practitioners are seeking to serve populations struggling with inactivity, overweight/obesity, aging and several controlled chronic conditions. Importantly, such groups of people represent a large part of the adult population not only in Southern Europe but also worldwide. However, despite the fact that gyms seem to be feasible settings for exercise in populations with cardiovascular risk factors in Italy [80], the current state of the physical activity, exercise and wellness sector does not appear well-equipped to offer an inclusive environment in order to support public health and meet its mission optimally [59].

### 4.7. The impact of technology

Certain changes in the fitness industry have been reported in the past decade due to the impactful role of social media and networks in the development of trends related to technology within the health and fitness sector [81]. Importantly, technology-oriented trends such as online personal training, mobile exercise apps, virtual training, online live and on-demand exercise classes and electrical muscle stimulation training were not included in the top 20 in all Southern European countries involved in the present survey. Only outcome measurements and wearable technology demonstrate some potential in Italy, Spain, Portugal and Greece, but not in Cyprus. This finding agrees with data recently published by similar studies carried out in several regions [32],[47]–[49]. The rapid digitalization and aggressive transformation of the traditional services mainly based on in-person fitness programs in a gym environment due to the COVID-19 pandemic may not have happened in an absolute way in all five countries involved in the Southern European survey. On the one hand, exercise professionals may understand the importance of applying technology for delivering their services under the urgent and challenging circumstances; however, the majority of them did not seriously follow the technology-oriented path in order to promote engaging fitness services and interactive user experience [82]. In summary, digital services do not seem to be high-priority fitness trends in the Southern European health and fitness sector. This outcome is aligned with the results presented by a Pan-European survey for 2020, 2021, 2022 and 2023 [47]–[50], indicating that trends related to technology are not widely established regionally among gym operators and practitioners. This observation cannot be explained here. However, further research is needed in this area to identify the potential factors that play a major role in the interaction among technology-oriented trends, job satisfaction and quality of life of fitness professionals and customer engagement in the Southern European physical activity, exercise and wellness industry.

### 4.8. COVID-19: A “game changer”

Considering that 2023 appears to be a critical transition and recovery period from the global epidemiological crisis due to COVID-19 [1],[3], it is important to detect the role of COVID-19 in the top trends of the physical activity, exercise and wellness industry. Notably, the industry's aggressive and rapid adaptation to an unprecedented business environment played a crucial role in the provision of fitness services among the masses globally. In all Southern European countries, fitness clubs closed for almost 6 months in both 2021 and 2022. When indoor fitness facilities reopened, they experienced numerous challenges and barriers due to rigorous hygiene protocols and restrictions with regard to the global pandemic. Such regulations discouraged customers from using the gym facilities, and more importantly, exercise behavior was modified for reasons related to safety and social distancing. This may explain the rising attractiveness of home exercise gyms and outdoor activities in all relevant studies conducted during the COVID-19 pandemic at global level [32],[47],[48]. However, outdoor activities are still popular in the Southern European region for 2023, demonstrating that walking, running and riding may be accessible and pleasant physical activity solutions. It would be interesting to investigate the evolution of all fitness trends highly impacted by COVID-19 in the future in order to identify the critical role of the pandemic crisis on the state of the physical activity, exercise and wellness sector in Southern Europe and other regions around the globe.

### 4.9. Strengths and limitations

The present survey, as an observational study, has several drawbacks, such as difficulties in determining causal effects, cohort differences and potential report biases. In particular, the absence of randomness in the samples and potential coverage errors appear to be the main limitations of this study. No incentive (financial or material) was offered to participants across all Southern European studies, which may be an additional tool for collecting more responses in this type of survey. On the other side, the replication of the methodology widely used by the ACSM and associates in relevant studies at national, regional and global levels seems to be the major strength of this study, highlighting a high degree of standardization that may lower potential bias frequently reported for research attempts of this kind. The large sample size (n = 2645) and the response rate (13.3%) in all involved national fitness surveys provide important summarized data for Southern Europe, which is an additional strength of the present survey. With the dissemination of the present results, Southern Europe is now included in a list of regions that regularly conduct this type of survey, allowing observations to be collected and compared over time. This type of observational study supports the evolution of services and products in the physical activity, exercise and wellness sector. Thus, it may be useful to continue with this research approach in Italy, Spain, Portugal, Greece and Cyprus in the coming years.

## 5. Conclusions

The results from this survey, focused on fitness trends in Southern Europe, may guide the physical activity, exercise and wellness sector in making critical business and professional development decisions. On average, personal training was the most attractive fitness trend in Southern Europe for 2023. This outcome is aligned with current results from a Pan-European survey [49] but not a global survey of fitness trends for 2023 [33]. This regional study delivers the opportunity to investigate useful comparisons among Italy, Spain, Portugal, Greece, Cyprus and other countries or regions in order to provide observations regarding the top health and fitness trends associated with various fitness services, products and programs worldwide [54]. Considering that the inactivity epidemic is on the rise not only in Southern Europe [83] but also worldwide [6], the aim of this regional cross-sectional study is to help all involved parties in the health and fitness industry on how to develop and apply safe, efficient and pleasant exercise solutions relevant to the most popular health and fitness trends. Given that the emerging development of the physical activity, exercise and wellness sector needs employers and fitness professionals to be continually modernized and to apply evidence-based practices within a multi-layered and challenging space [84], the translation of the present outcomes into high-quality services may enhance engaging exercise experiences for clients in the fitness market both locally and regionally. Lastly, further research should definitely help the investigation in this particular area, aiming to identify whether clients' opinions differ from the current findings in Southern Europe and other regions. Such an attempt may enhance the vital role of the physical activity, exercise and wellness sector, aiming to promote regular exercise as a top priority for the vast majority of the world's population through innovative and evidence-based services, programs and products.
